# Penetration Depth of Propylene Glycol, Sodium Fluorescein and Nile Red into the Skin Using Non-Invasive Two-Photon Excited FLIM

**DOI:** 10.3390/pharmaceutics14091790

**Published:** 2022-08-26

**Authors:** Mohammad Alhibah, Marius Kröger, Sabine Schanzer, Loris Busch, Jürgen Lademann, Ingeborg Beckers, Martina C. Meinke, Maxim E. Darvin

**Affiliations:** 1 Center of Experimental and Applied Cutaneous Physiology, Department of Dermatology, Venerology and Allergology, Charité–Universitätsmedizin Berlin, Corporate Member of Freie Universität Berlin and Humboldt-Universität zu Berlin, Charitéplatz 1, 10117 Berlin, Germany; 2 Department of Mathematics, Physics and Chemistry, Berliner Hochschule für Technik, Luxemburger Straße 10, 13353 Berlin, Germany; 3 Department of Pharmaceutics and Biopharmaceutics, Philipps University Marburg, 35037 Marburg, Germany

**Keywords:** skin barrier function, stratum corneum, epidermis, drug delivery, skin penetration, penetration pathways, fluorescence lifetime imaging

## Abstract

The stratum corneum (*SC*) forms a strong barrier against topical drug delivery. Therefore, understanding the penetration depth and pathways into the *SC* is important for the efficiency of drug delivery and cosmetic safety. In this study, TPT-FLIM (two-photon tomography combined with fluorescence lifetime imaging) was applied as a non-invasive optical method for the visualization of skin structure and components to study penetration depths of exemplary substances, like hydrophilic propylene glycol (*PG*), sodium fluorescein (*NaFl*) and lipophilic Nile red (*NR*) into porcine ear skin ex vivo. Non-fluorescent *PG* was detected indirectly based on the pH-dependent increase in the fluorescence lifetime of *SC* components. The pH similarity between *PG* and viable epidermis limited the detection of *PG*. *NaFl* reached the viable epidermis, which was also proved by laser scanning microscopy. Tape stripping and confocal Raman micro-spectroscopy were performed additionally to study *NaFl*, which revealed penetration depths of ≈5 and ≈8 μm, respectively. Lastly, *NR* did not permeate the *SC*. We concluded that the amplitude-weighted mean fluorescence lifetime is the most appropriate FLIM parameter to build up penetration profiles. This work is anticipated to provide a non-invasive TPT-FLIM method for studying the penetration of topically applied drugs and cosmetics into the skin.

## 1. Introduction

With an area of about 1.5–2 m^2^, the skin covers most of the body and contributes to the mechanical and biochemical defense system [1]. Acting as an effective barrier against pathogen penetration and solar light exposure, it protects the body against temperature changes and water loss [2,3,4]. Due to this function, the skin forms a barrier against topically applied pharmaceuticals and cosmetics, i.e., ointments, creams and drugs. The stratum corneum (*SC*) is the uppermost layer of the skin consisting of corneocytes embedded in the structurally organized lipid matrix [3,5] and forms the strongest non-homogenous in-depth barrier between body and environment [6,7]. Cosmetics like sunscreens and skin moisturizers act in the *SC* and should not permeate the skin barrier [8,9], while drugs must first cross the *SC* to reach the living cells of the viable epidermis [10]. To ensure the permeation through the *SC*, different methods are used [11] including the addition of penetration enhancers into the formulations [12]. In general, molecules which can pass the *SC* can usually penetrate into the deeper layers of the skin [13]. Therefore, to guarantee efficient drug delivery, it is important to investigate the penetration into the *SC*. The penetration depth depends on several factors, including skin health, age, and most importantly, the substances themselves. Molecule size, solvents, and the hydrophilicity or lipophilicity of the substances play a crucial role in penetration [10,14,15].

Beside human skin, different skin models have been used in penetration studies, i.e., porcine and murine skin [6,16,17], which resemble human skin in terms of morphology and properties like thickness, radical production, and components. Differences in the barrier-related parameters of the *SC* between porcine ear and human skin have been, however, shown by Choe et al. [18].

Different methods have been considered for cutaneous penetration studies in vivo. Besides taking biopsies and histological sections prepared from them [14], there are many invasive methods, e.g., microdialysis [15,19,20] and the suction blister method [21]. Among the most commonly used methods are the minimally invasive tape-stripping and cyanoacrylate-stripping techniques [22]. Since the invasive and minimally invasive methods can be painful, unsafe, not applicable for kinetic measurements, and not permissible according to ethical rules [23], the focus is always on the application of advantageous optical non-invasive techniques. Confocal laser scanning microscopy (LSM) uses the fluorescence property of certain substances and thus can visualize the skin structure and the presence of these substances in different skin layers [24,25]. The method is suitable for both in vivo and ex vivo investigations; however, it requires a fluorescence-labelled substance and is, therefore, not suitable for non-fluorescent substances [14]. Raman micro-spectroscopy measures the energy of changes in the vibrational states of molecules, thus providing characteristic information about the molecules present in the sample [26]. The strong superposition of substance- and skin-related Raman bands, as well as the long measurement time and the high price of the device are considered as the main disadvantages of this method [27]. Optical coherence tomography is not appropriate for penetration studies due to non-detectable changes in the optical properties of the skin and the investigated formulation [25]. Furthermore, none of the mentioned methods provides clear information about the penetration pathways due to resolution restriction.

In this work we present the application of two-photon excited fluorescence lifetime imaging (TPE-FLIM) as a non-invasive optical method for studying the penetration into the skin. It is a pixel-by-pixel time-resolved imaging method for visualizing skin components and applied substances based on the analysis of the two-photon excited fluorescence intensity and subsequent determination of TPE-FLIM parameters of fluorophores [28,29]. TPE-FLIM offers many advantages, e.g., unlike the fluorescence intensity the fluorescence lifetime does not depend on the fluorophore concentration but on its molecular environment [24,30]. The dependence on the molecular environment enabled an imaging of Ca^2+^ concentration in cells [31,32], mapping oxygen [33,34], pH [35,36,37] and temperature [38,39] in cells as well as monitoring the viscosity of the plasma membrane [40]. Furthermore, many endogenous fluorophores overlap fluorescence emission spectra but have different lifetimes; thus TPE-FLIM can separate those fluorophores depending on their characteristic lifetime regardless of their emission wavelength [24,41]. Another advantage is that one photon excitation depends linearly on the excitation energy, but TPE is a function of the square of this energy [24,42,43] and occurs only in the focal plane, where the photon density is sufficient for two-photon absorption [44]. Moreover, restricting photochemical interaction to the focal plane reduces autofluorescence photobleaching [45,46].

TPE-FLIM provides a powerful method for detecting chemical and physical changes in the molecules [41], thereby providing an excellent opportunity for fluorescence lifetime and the microscopic environment-dependent visualization of substances [47,48] and cells in the skin [49,50,51]. The method has been also used to determine the morphological differences between healthy and cancerous skin [52,53,54,55,56]. Thus, the TPE-FLIM method has proved to be well suited for dermatological and skin-physiological studies.

The application of TPT-FLIM for penetration studies in the skin have been presented, for example, on zinc oxide nanoparticles [57], antibiotic minocycline gel [58], an anti-inflammatory compound [59], and silver nanoparticles [29]. Nevertheless, this application of TPE-FLIM has so far been rarely used in practice. Therefore, this work aims to explore the efficiency of the TPT-FLIM method for penetration studies on further hydrophilic and lipophilic substances with and without their own fluorescence.

## 2. Materials and Methods

### 2.1. Porcine Skin Samples

In this study, fresh porcine ears were obtained from a local butcher a few hours after slaughter. The ears were cleaned with cold tap water and dried with soft paper tissue. For each experiment, six different ears were used. The hair was carefully removed using scissors so that the *SC* was not damaged, then the ears were stored in a fridge at a temperature of 4 °C until the next day. Measurements were performed on fresh ears within 48 h of delivery to exclude an influence on the penetration experiments of post-mortem changes [60]. On the measurement day, a 2 cm × 2 cm area was selected and marked on each ear for the application of 20 μL/cm^2^ of the examined substance. A finger massage was applied gently in circular motion for 1 min to ensure that the solution was homogeneously distributed over the entire area. After that, the treated and untreated skin samples were incubated for 30 min at a temperature of 32 °C, which mimics the in vivo conditions of human skin surface temperature [61,62]. After the incubation, the rest of the substances was removed from the skin surface using a soft dry tissue, then cleaned with wet tissue 3–5 times. A 1 cm × 1 cm area was excised from the skin for the TPT-FLIM measurements. For tape stripping, a 6 cm × 6 cm sample was treated with 72 μL of the examined solution for 30 min. Before pressing the tapes onto the skin, a small piece of approx. 1 cm × 1 cm was excised for the Raman micro-spectroscopic measurements.

### 2.2. Propylene Glycol (PG)

*PG* (1,2-propanediol-CH_3_CH(OH)CH_2_OH)) is a colorless viscous liquid and is used as a vehicle for drugs as well as as a hydrophilic penetration enhancer [63]. The action mechanism of *PG* on the skin has not yet been well understood [64]. *PG* is thought to act by dissolving keratin through binding at the hydrogen bonding sites. It may also act by interacting with the polar head groups of the intercellular lipids and can thus weaken the barrier function of the *SC* [10,65]. In this study, *PG* (SIGMA-Aldrich Chemie GmbH, Steinheim, Germany) was applied undiluted on the skin and incubated for 30 min at 32 °C. The pH value of the *PG* was 7.29 ± 0.04. *PG* has no TPE fluorescence at 760 nm excitation.

### 2.3. Sodium Fluorescein (NaFl)

*NaFl* (C_20_H_10_Na_2_O_5_) is an orange-red salt powder of very good solubility in water, which has the ability to bind proteins [66]. *NaFl* was selected because it is often used in dermatology as a marker substance for penetration measurements in vivo and ex vivo [13,67]. The fluorescence lifetime of *NaFl* is pH dependent and varies between 3770–4110 ps in a pH range of 6.5–8.6 [68]. For TPT-FLIM and LSM measurements, 1 mL of *NaFl* solution was prepared by diluting 10 μL of *NaFl* 2% *w*/*v* eye-drop solution (Bausch & Lomb GmbH, Berlin, Germany) in 990 μL of phosphate-buffered saline (*PBS*), whose pH value is 7.4. After TPE by 760 nm a fluorescence lifetime of *τ* = 4090 ± 50 ps was measured using single-exponential decay. For tape stripping and confocal Raman micro-spectroscopy, a *NaFl* solution (Fluoreszein SE Thilo^®^ eyedrops, 1.7 mg/mL, Alcon Pharma GmbH, Freiburg, Germany) was applied to the skin. The treatment time was 30 min at 32 °C for both of the *NaFl*-solutions.

### 2.4. Nile Red (NR)

*NR* was chosen due to its strong lipophilicity and its emission-dependence on the polarity of the environment [69]. For example, phospholipids show a red-shifted emission spectrum, while the neutral lipids such as cholesterol and triglycerides show a yellow-shifted spectrum [69,70]. Not only the emission but also the fluorescence lifetime of *NR* varies depending on the polarity of its environment. The fluorescence lifetime of *NR* was examined in different solvents [70,71]. Taking into consideration a strong occlusion and swelling of the *SC* induced by topically applied petrolatum [72], which results in the formation of a waterfront and the reduced penetration depth of lipophilic *NL* [73], paraffin oil was chosen as a solvent. To prepare the *NR* solution, 1 mg of *NR* powder (SIGMA-Aldrich Chemie GmbH, Steinheim, Germany) was dissolved in 1 mL of paraffin oil (SIGMA-Aldrich Chemie GmbH, Steinheim, Germany) and mixed using a vortex shaker for 15 min, followed by 15 min in ultrasonic bath under a temperature of 30 °C. At last, the skin was treated with 80 μL of *NR* for 30 min at 32 °C. The fluorescence lifetime of the solution was *τ* = 3176 ± 53 ps, determined using a single-exponential decay after a TPE at 760 nm. The solvent used (paraffin oil) is itself non-fluorescent, can be used as a skin moisturizer, and does not permeate the *SC*, and thus cannot enhance the penetration and reach the viable epidermis [74,75]. The control skin samples were treated with the vehicle of *NR*, which is paraffin oil, to ensure that both samples were examined under the same conditions.

### 2.5. Two-Photon Tomography in Combination with Fluorescence Lifetime Imaging (TPT-FLIM)

A two-photon tomograph (TPT, Dermainspect, JenLab GmbH, Jena, Germany) equipped with a tunable (710–920 nm) Ti:sapphire laser (Mai Tai XF, Spectra Physics, Milpitas, CA, USA) operated with a 100 fs pulse at a repetition rate of 80 MHz was used for the horizontal imaging of the skin based on TPE. A drop of immersion oil (Immersol™ 518F, Carl Zeiss Jena GmbH, Oberkochen, Germany) was placed between the objective and the objective ring with glass (No.1, 18 mm, VWR, Darmstadt, Germany) along with a drop of water between the glass and the skin to provide refraction-index matching. A bandpass filter (410–680 nm) was used to detect the fluorescence signal after TPE at a wavelength of 760 nm. In addition, the TPT has a 375–385 nm bandpass filter for the detection of the second-harmonic generation (SHG) signal. The lateral and axial resolutions of the TPT-FLIM are approximately 0.5 μm and 1.2–2.0 μm, respectively, with a horizontal scan field of up to 350 μm × 350 μm and a vertical field of up to 200 μm and a numerical aperture of the objective of 1.3 [76]. The acquisition time to record one image was 6.8 s with a scan field of 75 μm × 75 μm. Stack images were acquired at 4 μm increments from the skin surface to a depth of 32 μm. The laser power was adjusted experimentally depending on the fluorophore applied on the skin regarding the imaging depth (Table 1).

The TPE-FLIM data were processed and analyzed using SPCImage software version 8.4 (Becker & Hickl, Berlin, Germany). The fluorescence lifetime decay was determined in each pixel of the skin images using a bi-exponential function and the maximum likelihood estimation method. The fast lifetime and amplitude decay components are $\tau_{1},\;\alpha_{1}$; the slow ones are $\tau_{2},\;\alpha_{2}$. The amplitude-weighted mean fluorescence lifetime $\tau_{m}$ was defined as shown in Equation (1):

(1) $$\tau_{m}=\left(\alpha_{1}\tau_{1}+\alpha_{2}\tau_{2}\right)/\left(\alpha_{1}+\alpha_{2}\right)$$

The fluorescence lifetime decay was averaged over the 48 neighboring pixels using a binning value of 3. The TPE-FLIM parameters of each image were exported using SPCImage software and the data were analyzed using Microsoft Excel 2019 to create parameter-depth charts. The utilized TPT-FLIM was described in detail by our group [29,50].

### 2.6. Confocal Laser Scanning Microscopy

A confocal laser scanning microscope (VivaScope^®^ 1500, Multilaser, MAVIG, Munich, Germany) was used in fluorescent mode for one-photon excitation. The system is equipped with three laser diodes (488, 685 and 785 nm) and an objective with a numerical aperture of 0.9. In this study, the 488 nm wavelength was chosen to investigate the skin in the fluorescent mode. A drop of immersion oil (Crodamol STS, Croda Inc., Snaith, UK) was applied between the skin and the ring glass (adhesive window with crosshair, Lucid Vision Labs GmbH, Ilsfeld, Germany). Ultrasonic gel (Aquasonic 100, Parker laboratories Inc., Fairfield, CT, USA) was placed between the objective and the objective ring glass to maintain the optical contact. The utilized LSM system was described previously by our group [77,78].

Stack images (500 μm × 500 μm) were made at 1.5 μm increments from the skin surface down to approximately 80 μm depth. The laser power was fixed at 5 mW for the treated and untreated skin, aiming at comparing the fluorescence intensities. Using ImageJ 1.53K software (Wayne Rasband, National Institute of Health, Bethesda, MD, USA), an area of 188 μm × 188 μm was selected on a fixed position of the images from the sample surface down to approximately 80 μm. The mean gray values of the selected areas were measured and then averaged at the corresponding depth.

### 2.7. Tape Stripping (TS) and UV/VIS Spectroscopy Measurements

The TS method was used to study the penetration of *NaFl* into porcine ear skin. A 72 μL quantity of the *NaFl* was applied onto a 6 cm × 6 cm area of each skin sample for 30 min, then the rest of the solution was removed. Adhesive tapes (Tesa^®^, No. 5529, Beiersdorf AG, Hamburg, Germany) were pressed onto the skin using a rubber roller weight of 746 g and rolled 5 times without external pressure; then the tape was removed with one swift movement. Following the method described by Jacobi et al. [79], approx. 84% of the *SC* was removed after 30 tapes had been stripped from the same area.

The tape strips were cut to a size of 1.9 cm × 3.4 cm and the fluorescein on them was extracted by solving each of them in 6.46 mL ethanol (Uvasol^®^ Ethanol 99.9%, Merck KGaA, Darmstadt, Germany) using an ultrasonic bath, then purified by centrifuge (MR 1812, Jouan GmbH, Unterhaching, Germany). The extract was decanted in a UV cuvette using a pipette and the absorbance was measured by a spectroscope (Lambda 650S, PerkinElmer, Frankfurt/Main, Germany) at 480 nm for the detection of *NaFl*.

### 2.8. Confocal Raman Micro-Spectroscopy (CRM)

Raman spectra were recorded using a skin-composition analyzer (Model 3510 SCA, River D International B.V., Rotterdam, The Netherlands) and CRM measurements were performed on the same 6 porcine ear-skin samples which had been used for the UV/VIS spectroscopy measurements. The fingerprint Raman spectra (400–2000 cm^−1^) were recorded from the skin surface down to a depth of 40 μm with a step size of 2 μm. The acquisition time was 5 s/spectrum, the excitation wavelength was 785 nm, and the maximal power at the surface was set to 20 mW. For each skin sample, 10 different points were measured. The semiquantitative concentration profiles of *NaFl* in the *SC* were determined for each measurement point using the unconstrained multiple least square fit method (available in the SkinTools software developed by RiverD International B.V.) [80] and averaged for further analysis. The utilized CRM was described previously by our group [27].

## 3. Results

TPT was used to determine the thickness of the *SC* based on the appearance of cells attributed to the stratum granulosum. On average, this was 16.0 ± 3.3 μm, which is consistent with data from the literature [3,75,81]. Thus, 16 μm was considered in the following results as the *SC* thickness.

### 3.1. Penetration of PG

**Figure 1:**
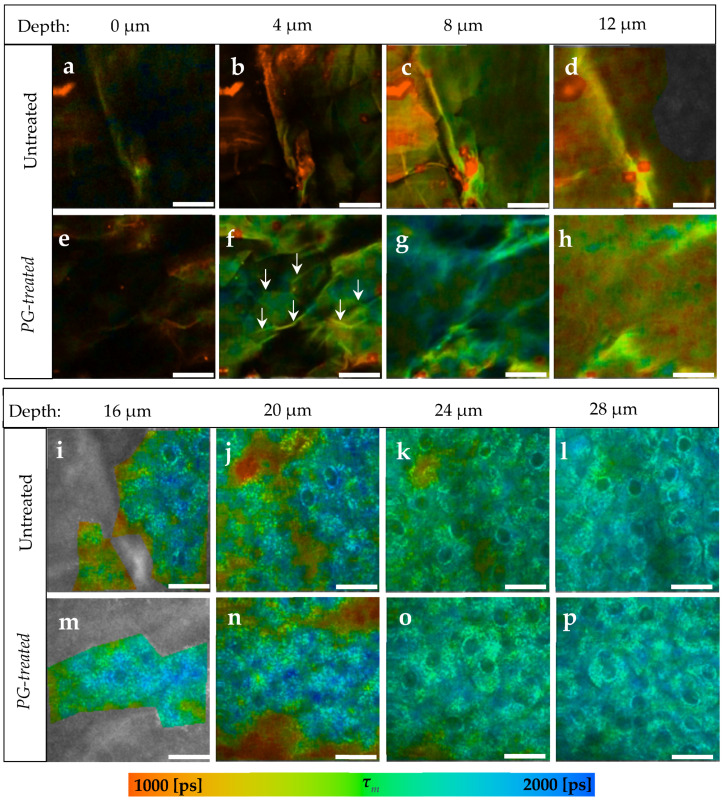
TPE-FLIM images depending on skin depth of *PG*-treated compared to untreated skin. (**a**–**d**) The *SC* of untreated skin sample; (**e**–**h**) The *SC* of *PG*-treated skin sample; (**f**) arrows refer to lines, which are assumed to be the areas between the corneocytes; (**i**–**l**) The viable epidermis of untreated skin. (**m**–**p**) The viable epidermis of *PG*-treated skin. In both samples there was no differences in the pseudocolors. *Τ_m_* was measured after excitation with 760 nm using TPT-FLIM and is shown by a pseudocolor scale of 1000–2000 ps. Scale bar: 20 μm. Acquisition time: 6.8 s. Excitation power is depth-dependent and shown in Table 1.

After applying 40 μL of *PG* on 2 cm × 2 cm skin sample, it was incubated for 30 min at a temperature of 32 °C. TPT-FLIM measurements were done starting from the skin surface down to a depth of 32 μm. *PG* has a pH = 7.30 ± 0.04, which is about 1 more than the skin surface of porcine ear [82]. Using pseudocolor scale of *τ_m_* = 1000–2000 ps, a comparison between the TPE-FLIM images for untreated and *PG*-treated skin is shown in Figure 1 for different depths.

Generally, an increase in *τ_m_* in the whole image down to a depth of 12 μm in *SC* was observed after the treatment with *PG*. In Figure 1f, *τ_m_* was about 334 ± 65 ps longer in the corneocytes, while in the areas between them (Figure 1f, arrows) the increase in *τ_m_* was about 280 ± 55 ps. Those areas were no more visible in the deeper *SC*, therefore it is assumed that those areas refer to the extracellular area separating the corneocytes. At 12 μm depth (Figure 1h), a small increase from *τ_m_* = 1134 ± 87 ps to *τ_m_* = 1278 ± 107 ps was measured. 

Compared to the untreated skin in Figure 1i–l, *τ_m_* of the viable epidermis was not affected after the treatment with *PG* (Figure 1m–p) showing a non-significant Δ*τ_m_* of only 14–40 ps.

TPE-FLIM parameters of both samples are presented in Table 2. *τ_1_*, *τ_2_,* and *τ_m_* increased in the *SC* after the treatment with *PG*, while the relative amplitudes were not affected since no additional fluorophore was applied on the skin. At 16 μm depth, the viable epidermis starts and the differences in TPE-FLIM parameters disappear.

**Figure 2:**
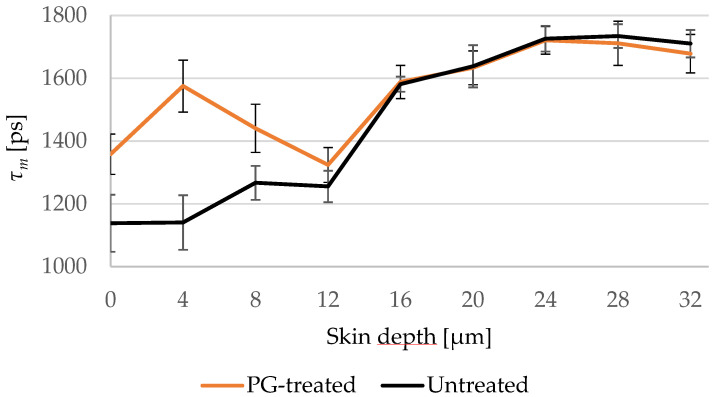
Changes of *τ_m_* of untreated (black line) and *PG*-treated (orange line) skin depending on the depth at 4 μm increment. *PG*-treated skin shows longer *τ_m_* down to 8 μm. *SC* thickness is 16.0 ± 3.3 μm. *N* = 6.

Furthermore, the penetration profile of *PG* in porcine ear skin using the average of *τ_m_* for the six ears is shown in Figure 2. The penetration profile exhibited the same results shown in Figure 1, where *PG* was only detectable in the *SC*.

### 3.2. Penetration of NaFl Using TPE-FLIM and LSM

**Figure 3:**
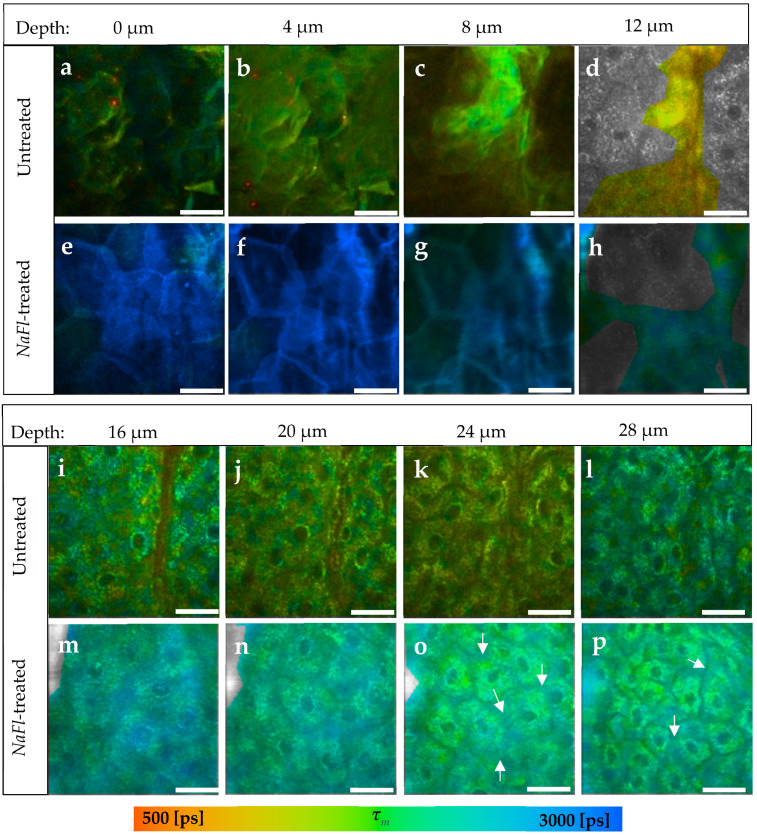
TPE-FLIM images of *NaFl*-treated compared to untreated skin at different depths. (**a**–**d**) The *SC* of the untreated skin; (**e**–**h**) The *SC* of *NaFl*-treated skin. The treated *SC* showed an average increase in *τ_m_* from *τ_m_* = 1522 ± 89 to *τ_m_* = 2725 ± 210 ps; (**i**–**l**) The viable epidermis of the untreated skin; (**m**–**p**) The viable epidermis of the *NaFl*-treated skin; (**o**,**p**) The white arrows refer to the extracellular areas between the keratinocytes. *τ_m_* was measured after an excitation with 760 nm using a TPT and is shown by a pseudocolor scale of 500–3000 ps. Scale bar: 20 μm. Acquisition time: 6.8 s. Excitation power is depth-dependent and shown in Table 1.

The fluorescence lifetime of the *NaFl* solution was measured by a single-exponential decay to be *τ* = 4090 ± 50 ps. The TPE-FLIM parameters of the untreated and the *NaFl*-treated skin were determined after a treatment time of 30 min and a TPE by 760 nm. Figure 3 shows the distribution of *NaFl* in the skin after treatment, where the *τ_m_* of the *SC* increases on average from *τ_m_* = 1522 ± 89 ps in the untreated sample (Figure 3a–d) to *τ_m_* = 2725 ± 210 ps in the treated one (Figure 3e–h).

Furthermore, *τ_m_* increased in the viable epidermis from *τ_m_* = 1667 ± 69 ps in the untreated (Figure 3i–l) to *τ_m_* = 2079 ± 42 ps in the *NaFl*-treated sample (Figure 3m–p). The TPE-FLIM parameters are shown in Table 3 for both skin samples. The fast and slow fluorescence lifetime components *τ_1_* and *τ_2_* had increased down to a depth of 16 μm, while *τ_m_* and the relative amplitude *α_2_* indicated an increase even in the viable epidermis till at least 32 μm depth. The fluorescence lifetime of the *NaFl* solution amounts to *τ* = 4090 ± 50 ps and is close to the *τ_2_* of the viable epidermis of the untreated skin, which varied in a range of *τ_2_* = (3553 ± 131)–(3956 ± 181) ps. Thus, *τ_1_* and *τ_2_* may not show a noticeable increment, but with their relative amplitudes *α_1_* and *α_2_* will, because they also represent the amount of the contributing fluorophores. This increase is therefore also detectable by *τ_m_* because it is amplitude-weighted lifetime.

**Figure 4:**
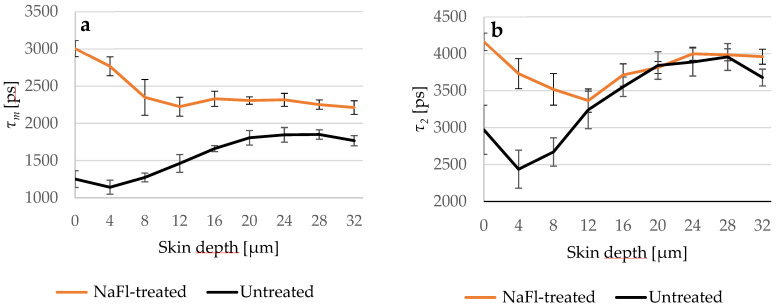
(**a**) Comparison between *τ_m_* of the untreated (black line) and *NaFl*-treated (orange line) skin depending on the depth; (**b**) *τ_2_* of both skin samples in different depths. *τ_2_* curves overlapped at 12 μm, while *τ_m_* of the *NaFl*-treated skin was longer at all measured depths. *SC* thickness is 16.0 ± 3.3 μm. *N* = 6.

Figure 4 presents the different penetration profiles of *NaFl* based on *τ_m_* and *τ_2_* and it shows also that the influence of *NaFl* on the viable epidermis using *τ_2_* as a representative TPE-FLIM parameter is not detectable.

**Figure 5:**
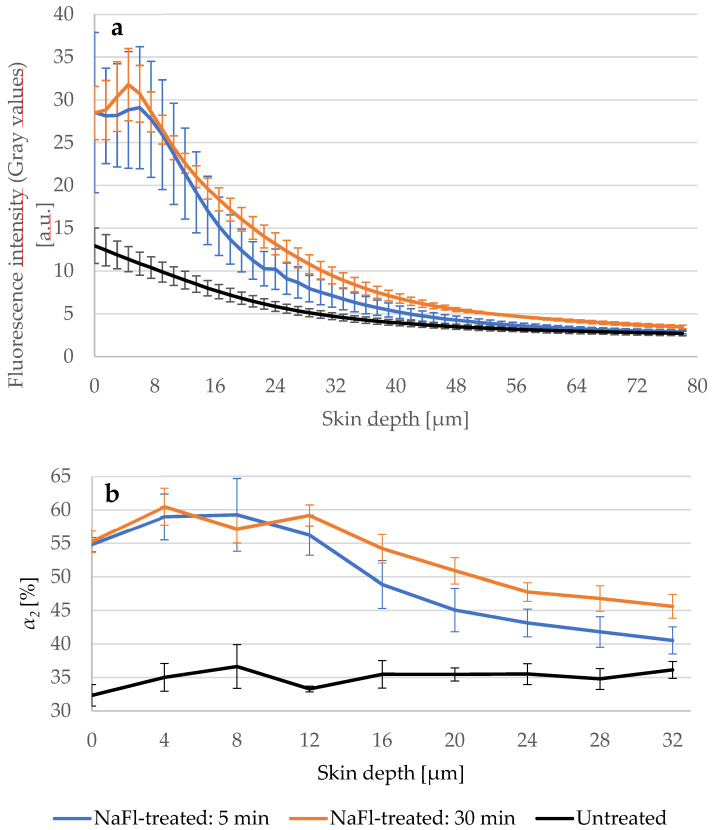
(**a**) LSM-fluorescence intensity of the skin after 5 (blue line) and 30 min (orange line) treatment with *NaFl* compared to untreated skin (black line). *SC*-thickness is 16.0 ± 3.3 μm. Excitation power was fixed at 5 mW. Gray values of LSM images were used as fluorescence intensities. Higher intensity was measured in the 30 min treated sample; (**b**) The relative amplitude of the slow lifetime component α_2_ increased after longer treatment time in the viable epidermis. TPE-excitation power shown in Table 1. *SC*-thickness is 16.0 ± 3.3 μm. *N* = 3.

To ensure that the penetration depth indicated by *τ_m_* is correctly determined, we treated three skin samples of different porcine ears with *NaFl* and measured the fluorescence intensity of the untreated and treated samples via LSM. The treatment time was 5 min and 30 min at 32 °C. Figure 5a shows that the fluorescence intensity in the viable epidermis of the 5- and 30-min treated skin was higher compared to the untreated skin. On the other hand, the 30-min treated sample showed a higher intensity than the 5-min treated sample indicating that more *NaFl* reached the viable epidermis after a longer treatment time. This result was confirmed by the relative amplitude *α_2_* (Figure 5b), which reflects the relative amount of the contributing fluorophores in *τ_2_*, which includes *NaFl* because it has a long fluorescence lifetime. In the viable epidermis from 16 μm to 32 μm depth, *α_2_* was on average 5.2 ± 0.2% higher in the 30-min treated than in the 5-min treated sample.

### 3.3. Penetration of NaFl Using CRM and TS

**Figure 6:**
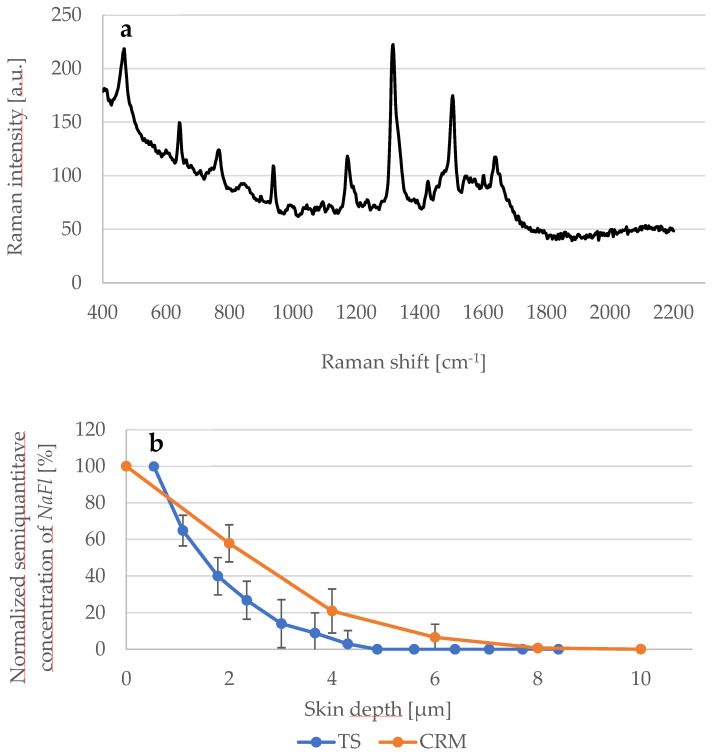
(**a**) Raman spectrum of *NaFl* excited at 785 nm; (**b**) Comparison between the detected *NaFl* concentration depending of the skin depth determined using CRM (orange line) and TS (blue line) methods. *N* = 6.

Further experiments were performed to study the penetration depth of *NaFl* using TS and CRM methods. The thickness of the *SC* in the examined skin samples was 20 μm. The Raman spectrum of the *NaFl* is presented in Figure 6a and the corresponding penetration profiles of *NaFl* in the skin are shown in Figure 6b. Results obtained using CRM show the exponential decay: at 2 μm depth, the concentration of *NaFl* decreased from 100% to 58 ± 10% and to 21 ± 12% at 4 μm depth. At 8 μm, no signal was detected.

Regarding TS and the UV/VIS spectroscopy measurements, the absorbance of the extracted *NaFl* was measured at 460 nm and the recorded value of the first TS was considered as 100%, so the absorbance from the next TS was calculated as a corresponding percentage value. Within the first 2 μm, the absorbance fell from 100% to 27 ± 10% and at 4 μm to 3 ± 7%. At 4.5 μm depth, no more absorbance was measured. As shown in Figure 6b, the value of the penetration-depth profile obtained by CRM is higher than by TS.

### 3.4. Penetration of NR

**Figure 7:**
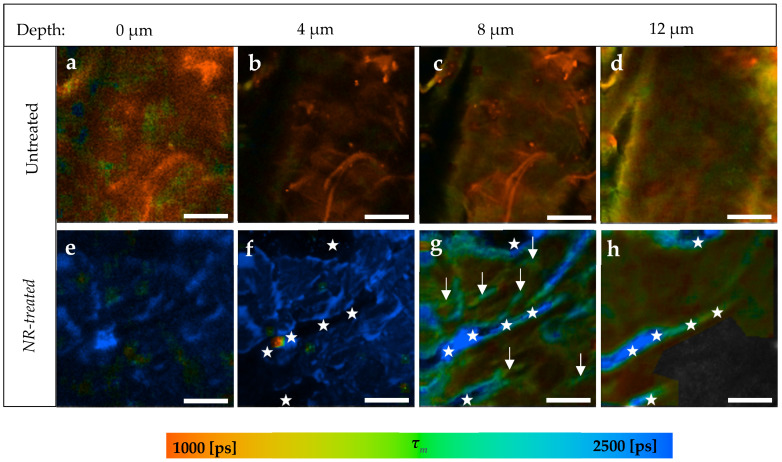
TPE-FLIM images of *NR*-treated compared to untreated skin depending on skin depth. (**a**–**d**) *SC* of untreated skin sample; (**e**–**h**) *SC* of *NR*-treated skin; (**g**) Arrows refer to areas with a longer *τ_m_* than the rest of the image indicating the presence of *NR*; (**f**) The stars refer to the background and stars in (**g**,**h**) refer to the skin surface because of skin furrow. *Τ_m_* was measured after an excitation with 760 nm using a TPT and is shown by a pseudocolor scale of 1000–2500 ps. Scale bar: 20 μm. Acquisition time: 6.8 s. Excitation power is depth-dependent and shown in Table 1.

In the last part of this study, the penetration of the lipophilic *NR* in the skin was investigated. The prepared *NR* solution had a fluorescence lifetime *τ* = 3176 ± 53 ps calculated by single exponential decay after TPE at 760 nm. Figure 7a–d presents an example of the *SC* of an untreated skin sample compared to an *NR*-treated sample (Figure 7e–h) taken from the same porcine ear.

*NR* was found to penetrate only in the *SC* without being able to reach the viable epidermis. In the first 4 μm (Figure 7a,b,e,f), *NR* was distributed in the *SC*, inducing an average increment of about *Δτ_m_* = 1963 ± 92 ps. Following this, the presence of *NR* was limited in specific areas of *SC* (Figure 7g, arrows), where *τ_m_* = 1926 ± 29 ps, while the rest of the same image of treated skin showed *τ_m_* = 1232 ± 35 ps.

The stars in Figure 7f refer to the background, as they are located on the black areas of the image. In Figure 7g,h, those areas are colored blue, because *NR* appears, thereby referring to the skin surface due to the furrows. Thus, stars’ locations were not considered as having been penetrated by *NR* at the corresponding depth. 

The TPE-FLIM parameters are shown in Table 4. Significant differences in *α_1_*, *α_2_,* and *τ_m_* were detected down to a depth of 12 μm, but only to an 8 μm depth in *τ_1_* and *τ_2_*. The untreated skin had a fluorescence lifetime *τ_2_* = 3155 ± 192 ps at 12 μm depth, which is similar to the fluorescence lifetime of *NR* solution with *τ* = 3176 ± 53 ps. Therefore, the presence of *NR* at this depth did not induce changes in *τ_2_*. However, the relative amplitude α_2_ increased at 12 μm from 32.4 ± 0.9% to 49.0 ± 1.6%. The viable epidermis started at around 16 μm, where all of the TPE-FLIM parameters were comparable in both skin samples.

**Figure 8:**
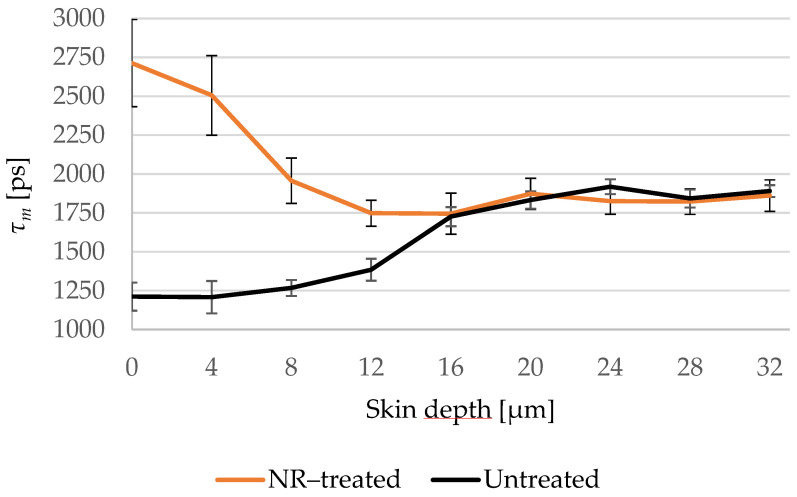
Changes of *τ_m_* of the *NR*-treated (orange line) compared to the untreated (black line) skin depending on the depth at 4 μm increments. *τ_m_* of the *NR*-treated skin decreases in the deep *SC* and overlaps with the untreated skin in the viable epidermis. *SC* thickness is 16.0 ± 3.3 μm. *N* = 6.

The mean fluorescence lifetime *τ_m_* was chosen to create the penetration profile of *NR* in the skin and Figure 8 shows that *τ_m_* of the treated and untreated samples overlaps in the viable epidermis and hence *NR* did not permeate the *SC*.

## 4. Discussion

The penetration depth of three different substances—*PG*, *NaFl* and *NR*—into porcine ear skin ex vivo was evaluated non-invasively using TPE-FLIM. It was possible to study the penetration of a non-fluorescent substance like *PG* taking advantage of the dependence of TPE-FLIM on the environmental parameter, which in this study was the pH value.

*PG* shows no fluorescence at 760 nm, but its pH value is about 1 higher than the *SC* [83,84]. Thus, the pH-dependence of the fluorescence lifetime [35,36,37,85] was taken into account, aiming at proving the presence of *PG* indirectly through the influence of its pH value on the fluorescence lifetime of the endogenous fluorophores. Therefore, the FLIM-parameters *τ_1_, τ_2_,* and *τ_m_* increased, which was shown in Table 2. Based on the TPE-FLIM images in Figure 1, the increase in *τ_m_* was detected in the whole space of the *SC* images, thus *PG* affected the corneocytes and the extracellular region. This leads to the conclusion that *PG* penetrated transcellularly, passing the intra- and the intercellular penetration pathways in the *SC*, which is typical for lipophilic substances [1,65,86]. Nevertheless, *PG* could not be detected anymore in the viable epidermis, which starts at around 16 μm depth. Mujica Ascencio et al. [63] showed by using multivariate analysis of Raman spectra that *PG* can permeate the *SC* and reach the depth of max. 22.0 μm in the skin, where the thickness of *SC* was ≈18 μm. This penetration depth could not be precisely detected using TPE-FLIM because *PG* and the viable epidermis share the same pH value of pH ≈ 7.4 [82] and thus cannot induce further changes in its fluorescence lifetime, which can be explained by the limited sensitivity of TPE-FLIM method to the non-fluorescent *PG*.

For fluorescent dyes like *NaFl* and *NR* with *τ=* 4090 ± 50 ps and *τ=* 3176 ± 53 ps, respectively, *τ_m_* was more suitable than *τ_1_* and *τ_2_* for studying the penetration depth, due to the similar values of *τ_2_* in the viable epidermis and the fluorescence lifetime of both examined substances. In this case, the lifetime components of the untreated skin will overlap with the components of the treated skin and the presence of the exogenous fluorophore is detectable only when the relative amplitude is taken into consideration because this amplitude is sensitive to the relative amount of the fluorophores. The application of TPE-FLIM to study the penetration of zinc oxide nanoparticles was presented by Roberts et al. [57,87] and showed the limitation of TPE-FLIM due to the overlapping of the *τ_1_* and *τ_2_* of zinc oxide with the autofluorescence lifetime of the endogenous fluorophores. As a solution, they used appropriate emission-bandpass filters to exclude the emission and lifetime of specific endogenous fluorophores. In this study, we show that this limitation can be overcome without a bandpass filter by taking the relative amplitudes into account, because they reflect the proportion of *τ_1_* and *τ_2_*. Thus, *τ_m_* can distinguish between two samples that have similar fast and slow lifetime components but in different fractions.

*NaFl* reached the viable epidermis and diffused into it. This result was presented by the TPE-FLIM parameters shown in Table 3, excluding *τ_1_* and *τ_2_* for the reason mentioned above. The penetration depth was confirmed by fluorescence-intensity analysis using LSM, where the gray values of the intensity images were considered as fluorescence intensity. The fluorescence intensity was measured after 30 min of treatment with *NaFl* and was, e.g., at 40.5 μm depth *I _30 min-treated_* =6.8 ± 0.5 a.u., while in the untreated skin *I _untreated_*= 3.9 ± 0.3 a.u. which refers to the presence of *NaFl* in the stratum spinosum. Furthermore, the same measurement was repeated on each skin sample after 5 min of treatment and the fluorescence intensity was *I _5 min-treated_* =5.2 ± 0.7a.u., which is lower compared to 30 min of treatment but still higher than in the untreated skin. Consequently, *NaFl* is able to penetrate at least 40.5 μm into the epidermis within only 5 min, and more *NaFl* diffuses into the viable epidermis after 30 min. As the relative amplitude *α_2_* reflects the relative amount of the contributing fluorophores in *τ_2_*, the parameter *α_2_* was measured in the three samples. Again, more *NaFl* was detected in the viable epidermis after 30 min than after 5 min which is confirmed by LSM data.

The CRM data showed a smaller penetration depth of approximately 8 μm. However, with only approximately 4 μm, the penetration depth using TS and UV/VIS spectroscopy was the smallest compared to the other methods. According to these CRM and TS results, it can be concluded that *NaFl* does not permeate the *SC*, while it was detectable in the stratum spinosum down to at least 32 μm using TPE-FLIM, and until at least 40 μm using LSM. The differences in the results for TS, CRM, TPE-FLIM, and LSM could be attributed to differences in the sensitivity of each method. According to O’goshi and Serup [66], *NaFl* can hardly permeate through the *SC*. Further information about the penetration depth of *NaFl* applied topically onto the skin could not be found in the literature. However, in this study, we demonstrated deeper penetration into the viable epidermis using TPT-FLIM and LSM.

The hydrophilic *NaFl* penetrated via the transcellular pathway in the *SC*, as all points on the TPE-FLIM images of the *SC* (Figure 3e–h) indicate an increase in *τ_m_*. In the viable epidermis, the structure of the keratinocytes was more apparent, where the lifetime of the intercellular region between the keratinocytes had increased more with *τ_m_* = 2169 ± 20 ps (Figure 3o–p, arrows) compared to the keratinocytes with *τ_m_* = 1918 ± 21 ps. This leads to the assumption that *NaFl* crossed the lipophilic barrier of the *SC* transcellularly and intercellularly and diffused into the hydrophilic viable epidermis, with a higher presence in the intercellular space of the viable epidermis. The intercellular penetration pathway of *NaFl* in the human viable epidermis was shown previously by Roberts et al. [87] who injected *NaFl* into the skin and investigated it using fluorescence-intensity images after TPE at 920 nm.

Based on the analysis of *τ_m_*, *NR* was found not to permeate the *SC*. It is known that the fluorescence lifetime of *NR* depends on the polarity of its environment [70] as well as on the kind of lipids it is binding to. For example, cholesterol and phospholipids induce a longer lifetime of 4200 ps, while triglycerides induce a shorter lifetime of approx. 3000 ps [70,88,89]. The lipid content in the viable epidermis is small compared to the *SC* [90], but even if *NR* was present in the viable epidermis, it should have bound to those lipids and therefore induce a longer *τ_m_* compared to the untreated skin. Thus, we conclude that *NR* did not reach the viable epidermis. In addition, the viable epidermis being a hydrophilic layer [91] with a water content of 60–70% [92] should prevent *NR* from penetration into the viable epidermis. Comparing to the literature, the penetration depth of *NR* solved in *PG* [93] as well as of *NR* gel [94] was examined using LSM. Both studies showed the same results: that *NR* does not reach the viable epidermis.

TPE-FLIM images of the *NR*-treated *SC* showed at 8 μm depth (Figure 7g, arrows) areas with longer *τ_m_* = 1926 ± 92 ps compared to the rest of the image with *τ_m_* = 1232 ± 35 ps. It is assumed that those areas refer to the lipid matrix in the intercellular penetration pathway, because the intercellular lipids should bind the *NR* molecules as shown by Talreja et al. [95] due to the strong lipophilicity of *NR*, thereby inducing a longer *τ_m_* as we observed in this study. On this basis it can be summarized that *NR* penetrates into the *SC* through the lipophilic intercellular pathway, but does not permeate the *SC*.

## 5. Conclusions

The outcome of this study demonstrates that TPE-FLIM can be a powerful non-invasive method for skin-penetration studies. We have shown that TPE-FLIM is not limited to fluorescent dyes, but can also indirectly detect non-fluorescent substances depending on their influence on the molecular environment of the skin’s endogenous fluorophores. However, this influence may disappear if skin parameters, e.g., the pH differences between the *SC* and the viable epidermis, change. Furthermore, the study showed how the choice of the TPE-FLIM parameters can affect the evaluation of the penetration depth of fluorescent dyes, i.e., if the examined substance has a fluorescence lifetime, which is similar to one of the fluorescence-lifetime components (*τ_1_* or *τ_2_*) and therefore the amplitude-weighted *τ_m_* is proposed as a representative parameter for the penetration profile in the skin. Based on this study, we found that both *PG* and *NR* cannot permeate the *SC* and may penetrate at least 12 μm into the *SC* (*SC* thickness is 16.0 ± 3.3 μm), while *NaFl* reaches the viable epidermis down to a depth of at least 40 μm. In contrast, TS and CRM detected *NaFl* only in the superficial *SC* depth, and the observed differences are explained by the different sensitivity of the applied methods. *PG* and *NaFl* penetrate transcellularly as they are hydrophilic, and *NR* intercellularly due to its lipophilic property.

## Figures and Tables

**Table 1:** Laser power (TPE at 760 nm) for the fluorophore-treated skin depending on the depth.

Skin Depth [μm]	Excitation Power [mW]			
	*PG* and Untreated Skin	*NaFl*	*NR*	Paraffin Oil
0	10	1	5	10
10	18	2	12	15
20	23	10	20	20
30	33	23	28	28
40	40	33	40	40

**Table 2:** TPE-FLIM parameters of untreated and *PG*-treated skin. Mean ± SEM. *N* = 6.

	Untreated Skin					*PG*-Treated Skin				
Depth [μm]	*τ*_1_ [ps]	*τ*_2_ [ps]	*τ_m_* [ps]	*a*_1_ [%]	*a*_2_ [%]	*τ*_1_ [ps]	*τ*_2_ [ps]	*τ_m_* [ps]	*a*_1_ [%]	*a*_2_ [%]
0	463 ± 46	2628 ± 184	1138 ± 91	65.7 ± 0.9	34.34 ± 0.9	599 ± 35	2955 ± 190	1358 ± 64	63.8 ± 2.2	36.2 ± 2.2
4	455 ± 56	2501 ± 253	1141 ± 87	65.5 ± 2.0	34.5 ± 2.0	671 ± 59	3489 ± 288	1575 ± 83	65.9 ± 2.8	34.1 ± 3.2
8	516 ± 34	2819 ± 186	1267 ± 54	66.3 ± 1.6	33.7 ± 1.6	574 ± 57	3236 ± 283	1441 ± 77	66.3 ± 2.8	33.7 ± 2.8
12	491 ± 31	2816 ± 152	1256 ± 50	66.5 ± 1.2	33.5 ± 1.2	518 ± 24	3062 ± 147	1324 ± 55	67.8 ± 0.8	32.2 ± 0.8
16	605 ± 11	3570 ± 86	1581 ± 24	66.5 ± 1.5	33.5 ± 1.5	606 ± 35	3620 ± 179	1588 ± 53	67.0 ± 1.0	33.0 ± 1.0
20	618 ± 28	3611 ± 149	1638 ± 67	65.5 ± 1.0	34.5 ± 1.0	616 ± 21	3690 ± 109	1633 ± 54	66.8 ± 0.5	33.2 ± 0.5
24	644 ± 19	3846 ± 100	1726 ± 41	65.8 ± 0.9	34.2 ± 0.9	651 ± 17	3900 ± 99	1721 ± 44	66.9 ± 1.2	33.1 ± 1.2
28	656 ± 22	3882 ± 156	1735 ± 38	66.1 ± 1.3	33.9 ± 1.3	646 ± 18	3843 ± 127	1712 ± 70	66.6 ± 0.7	33.4 ± 0.7
32	654 ± 23	3801 ± 96	1710 ± 44	66.1 ± 0.7	33.9 ± 0.7	636 ± 16	3743 ± 112	1679 ± 61	66.4 ± 0.9	33.6 ± 0.9

**Table 3:** TPE-FLIM parameters of untreated and *NaFl*-treated skin. Mean ± SEM. *N* = 6.

	Untreated Skin					*NaFl*-Treated Skin				
Depth [μm]	*τ*_1_ [ps]	*τ*_2_ [ps]	*τ_m_* [ps]	*a*_1_ [%]	*a*_2_ [%]	*τ*_1_ [ps]	*τ*_2_ [ps]	*τ_m_* [ps]	*a*_1_ [%]	*a*_2_ [%]
0	555 ± 73	2970 ± 332	1253 ± 113	67.7 ± 1.6	32.3 ± 1.6	1655 ± 168	4164 ± 118	3005 ± 109	44.8 ± 1.6	55.2 ± 1.6
4	441 ± 55	2437 ± 257	1143 ± 94	65.0 ± 2.1	35.0 ± 2.1	1325 ± 177	3730 ± 203	2766 ± 126	39.5 ± 2.8	60.5 ± 2.8
8	471 ± 36	2671 ± 193	1275 ± 58	63.4 ± 3.3	36.6 ± 3.3	849 ± 228	3518 ± 214	2349 ± 240	42.9 ± 2.0	57.1 ± 2.0
12	585 ± 69	3241 ± 254	1463 ± 120	66.7 ± 0.4	33.3 ± 0.4	605 ± 84	3366 ± 157	2225 ± 127	40.9 ± 1.6	59.1 ± 1.6
16	599 ± 15	3553 ± 131	1661 ± 40	64.5 ± 2.1	35.5 ± 2.1	732 ± 63	3713 ± 151	2330 ± 101	45.9 ± 1.9	54.2 ± 2.1
20	665 ± 49	3841 ± 187	1807 ± 97	64.6 ± 1.0	35.4 ± 1.0	767 ± 25	3813 ± 81	2306 ± 49	49.1 ± .19	50.9 ± 1.9
24	691 ± 44	3888 ± 189	1847 ± 97	64.5 ± 1.6	35.5 ± 1.6	792 ± 27	3998 ± 89	2316 ± 87	52.2 ± 1.4	47.8 ± 1.4
28	690 ± 32	3956 ± 181	1852 ± 64	65.2 ± 1.6	34.8 ± 1.6	756 ± 16	3984 ± 81	2252 ± 63	53.2 ± .19	46.8 ± 1.9
32	671 ± 30	3678 ± 114	1768 ± 69	63.9 ± 1.3	36.1 ± 1.3	761 ± 30	3960 ± 102	2214 ± 90	54.4 ± 1.8	45.6 ± 1.8

**Table 4:** TPE-FLIM parameters of untreated and *NR*-treated skin. Mean ± SEM. *N* = 6.

	Untreated Skin					*NR*-Treated Skin				
Depth [μm]	*τ*_1_ [ps]	*τ*_2_ [ps]	*τ_m_* [ps]	*a*_1_ [%]	*a*_2_ [%]	*τ*_1_ [ps]	*τ*_2_ [ps]	*τ_m_* [ps]	*a*_1_ [%]	*a*_2_ [%]
0	498 ± 42	2723 ± 155	1212 ± 90	65.2 ± 1.3	34.8 ± 1.3	1268 ± 250	4706 ± 538	2713 ± 281	49.0 ± 2.2	51.0 ± 2.2
4	469 ± 45	2485 ± 169	1208 ± 104	63.1 ± 0.9	36.9 ± 0.9	1123 ± 209	3586 ± 245	2506 ± 256	45.2 ± 1.5	54.8 ± 1.5
8	501 ± 32	2772 ± 185	1267 ± 51	65.5 ± 1.4	34.5 ± 1.4	541 ± 75	3111 ± 150	1957 ± 146	44.7 ± 3.0	55.3 ± 3.0
12	559 ± 38	3155 ± 192	1384 ± 71	67.6 ± 0.9	32.4 ± 0.9	510 ± 23	3065 ± 100	1748 ± 83	51.0 ± 1.6	49.0 ± 1.6
16	618 ± 22	3755 ± 153	1726 ± 62	64.3 ± 0.6	35.7 ± 0.6	602 ± 45	3587 ± 211	1745 ± 132	62.6 ± 1.4	37.4 ± 1.4
20	656 ± 25	3867 ± 138	1833 ± 55	62.9 ± 0.7	37.1 ± 0.7	665 ± 23	3943 ± 135	1873 ± 100	63.1 ± 1.6	36.9 ± 1.6
24	695 ± 15	4059 ± 105	1919 ± 48	63.3 ± 0.4	36.7 ± 0.4	663 ± 13	3808 ± 79	1825 ± 84	62.9 ± 1.7	37.1 ± 1.7
28	665 ± 21	3853 ± 142	1842 ± 58	62.7 ± 0.6	37.3 ± 0.6	664 ± 19	3844 ± 123	1822 ± 82	63.4 ± 1.6	36.6 ± 1.6
32	698 ± 18	4020 ± 88	1890 ± 38	63.9 ± 0.5	36.1 ± 0.5	698 ± 24	3964 ± 165	1861 ± 101	64.4 ± 1.2	35.6 ± 1.2

## Data Availability

The data are available from the corresponding author upon reasonable request.
